# Overview of Image Datasets for Deep Learning Applications in Diagnostics of Power Infrastructure

**DOI:** 10.3390/s23167171

**Published:** 2023-08-14

**Authors:** Bogdan Ruszczak, Paweł Michalski, Michał Tomaszewski

**Affiliations:** Department of Computer Science, Opole University of Technology, 45-758 Opole, Poland

**Keywords:** machine learning, dataset, power lines, deep learning, object detection

## Abstract

The power sector is one of the most important engineering sectors, with a lot of equipment that needs to be appropriately maintained, often spread over large areas. With the recent advances in deep learning techniques, many applications can be developed that could be used to automate the power line inspection process, replacing previously manual activities. However, in addition to these novel algorithms, this approach requires specialized datasets, collections that have been properly curated and labeled with the help of experts in the field. When it comes to visual inspection processes, these data are mainly images of various types. This paper consists of two main parts. The first one presents information about datasets used in machine learning, especially deep learning. The need to create domain datasets is justified using the example of the collection of data on power infrastructure objects, and the selected repositories of different collections are compared. In addition, selected collections of digital image data are characterized in more detail. The latter part of the review also discusses the use of an original dataset containing 2630 high-resolution labeled images of power line insulators and comments on the potential applications of this collection.

## 1. Introduction

The main idea behind this paper is to collect datasets related to power lines which are dedicated, among other things, to improving the maintenance processes. The article tries to fill the gap in the field of datasets usable for training deep learning models for the power industry. The overview provided here could be used as a source list for other researchers interested in training their own deep learning models with open source datasets.

**Figure 1:**
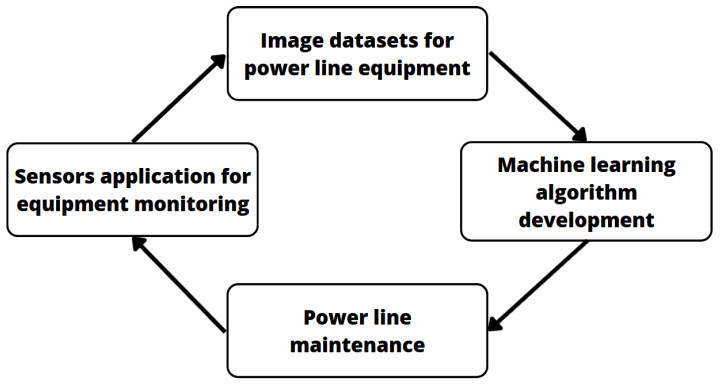
Overview of processes related to power line maintenance that are supported by algorithms trained on dedicated datasets.

Many researchers have dedicated their time to providing powerful algorithms and applications for the power industry. They aim to provide solutions for monitoring and supporting maintenance processes, and the current boom in deep learning methods has made this possible. However, such an approach usually requires a large amount of good quality and well-labeled data to be exposed to the algorithm training process (related processes are depicted in Figure 1).

The main goal of this article is to present the process of creating datasets used in training deep learning models in the context of atypical objects such as high-voltage power lines. The process of creating datasets for extensive objects that are critical elements of a country’s economy encounters several issues, as indicated in the presented paper. To achieve this, the following several conditions are recommended:

- Comprehensiveness assessment: To evaluate the comprehensiveness of existing datasets available in the field of power line maintenance. This objective aims to identify any gaps or limitations in the current datasets and highlight areas where additional data collection may be needed.
- Data comparison: To compare multiple datasets from different sources or studies, aiming to identify similarities, differences, and potential inconsistencies. This objective can help researchers to understand the strengths and weaknesses of different datasets and choose the most appropriate one for their analysis.
- Data accessibility and open data evaluation: To assess the availability and accessibility of datasets for the research community. This objective is relevant for promoting open data practices and making datasets easily accessible to other researchers.
- High-quality evaluation: To assess the quality and reliability of datasets by examining their data sources, collection methods, and data processing techniques. This objective helps ensure that the datasets used in research are of high quality and suitable for analysis.

For the deep learning algorithms that will later be used for classification or detection, these data are usually images or videos (in other words, long sequences of images). Such data collections are the ones of interest for this paper. What is reported by many researchers who prepare and share their training datasets (i.e., [1]) is that there are two most important things to consider: (i) having a sufficient number of images, a number that corresponds to the complexity of the problem; and (ii) a good quality of the reference information. These two determine the success of the latter application. If the intended process requires distinguishing many different types of power line equipment, the dataset must contain many images of all types of such equipment. If the system is aimed at identifying damaged elements, it forces their enumeration in the developed collection.

Power lines are a specific object that generates additional complications during data collection, the most problematic of which are safety concerns (working with power lines and related infrastructure can be dangerous; it involves working at heights and in close proximity to high-voltage electrical systems, posing risks to workers and vehicles collecting data); private property and permits (power lines often cross private property, and it can be difficult to obtain permission from landowners or utility companies to access their land for data collection); variability in infrastructure (power line infrastructure can vary widely in design, age, and materials used; creating a dataset that covers this variability is essential for it to be comprehensive and applicable to different scenarios), environmental factors (data collection for power line datasets can be affected by various environmental factors such as weather conditions, e.g., rain, snow, or extreme temperatures), and natural events (e.g., storms or earthquakes, which can complicate the process).

Equally important is the precise reference information for each data sample. Often it is quite easy to depict many parts of a power line, but later they must be carefully studied and, as in the case of detection tasks, require precisely marked classes on each image. This usually triggers a long process of dataset preparation and review, which for some advanced or unique components may depend on external domain expertise.

Nowadays, the field of power line dataset generation is evolving and researchers are facing several challenges to improve data quality, accessibility, and utility. In our view, the most important current research challenges are:

- Data fusion and integration: Researching methods to combine data from multiple sensors and sources to create more informative datasets. Integrating data from different types of sensors, such as visual cameras, thermal imaging, and acoustic sensors, can provide a more comprehensive understanding of power line conditions,
- Real-time analysis and decision making: Advancing real-time data analytics to enable proactive decision making and predictive maintenance for power line assets. This includes developing algorithms that can quickly process large amounts of data to identify potential problems and optimize grid performance,
- Automated data collection: Developing advanced techniques for automated data collection from power line assets using drones, satellites, LiDAR, or other remote sensing technologies. Automation can reduce human intervention, improve data coverage, and enable more frequent data updates.

Current research, e.g., [2], provides many examples of higher performance for object detection applications in the power line industry that make extensive use of image datasets, sometimes even with a limited amount of input data. The tests mentioned in [2] comment on several popular deep neural networks for processing images acquired from diagnostic videos recorded during inspection flights conducted along high-voltage power lines. In detail, it reports the most beneficial results for the faster region convolutional neural network (Faster R-CNN; achieving the highest AP above 0.8). The same study also mentions the need to use a dataset of appropriate size and the influence of this on the final score of the algorithm.

However, there are many other researchers who, in addition to presenting an already working algorithm, also emphasize the very high importance of the dataset and the high difficulty of its preparation, and suggest the necessity of obtaining more training material. These postulates can be found, among others, in [3], who describes the classification of transmission line scenes, in both [4,5,6,7] who present methods for insulator detection, or for fault detection based on aerial images of insulators [8,9,10,11].

Fortunately, instead of performing a costly and demanding dataset development every time images are involved, one could choose to adapt one of the existing sets. This paper aims to review the most productive collections that could be productive for researchers and companies interested in publishing algorithms for power line industry applications.

First, we checked the background of this topic and decided to analyze the popularity of this subject. The following terms were investigated to check their popularity in terms of the number of indexed journal and conference publications and their impact (measured by the number of citations): datasets for power lines, for conductors (or wires), for power line insulators, or for detection or for classification, and for the same keywords. This should be interpreted with caution since the terminology in the field varies from researcher to researcher (which is why we have verified the alternative combinations of the searched terms), but it still indicates a rapid increase in the field and its development.

**Figure 2:**
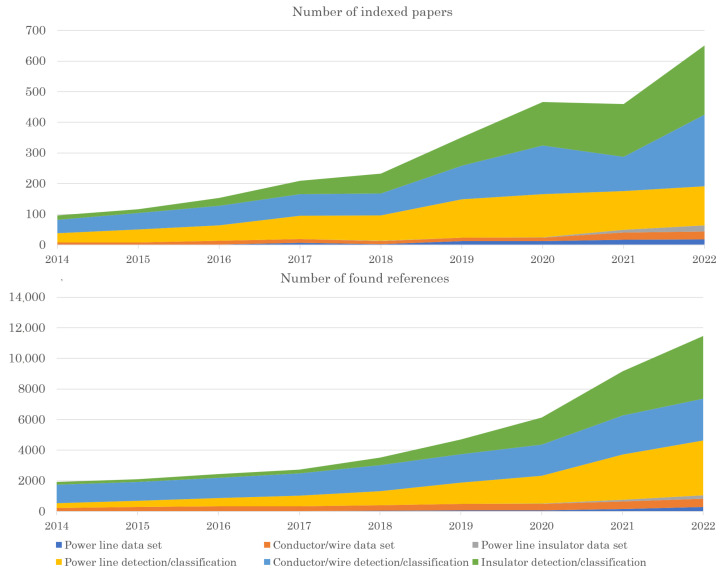
The related research metrics investigation. Figures were prepared using data from the *dimensions.ai* web service of Digital Science & Research Solutions, Inc., London, United Kingdom (accessed on 3 January 2023).

The investigation revealed that the number of papers discussing the datasets for the power line equipment has exceeded 220 in the last few years. If we also include in the search the number of papers dedicated to the widespread applications of these datasets, such as classification or detection tasks for the power lines, conductors, or insulators, we obtain more than 580 papers published in 2022 alone. The summary of these numbers is shown in Figure 2. We used the same queries to count the number of citations for this field and it also suggests an increase in referencing research.

## 2. Power Line Elements Datasets Review

The following part of the paper consists of two subsections. Section 2.1 provides an overview of the datasets with an indication of their main features and discusses a compilation of studies that use such data to develop algorithms and applications for the power industry. Section 2.2 includes a consideration of an example collection, which details the structure and potential applications of such collections, and deals with the detection of power insulators in images.

### 2.1. The Review of Datasets Consisting of Images of Various Power Line Equipment

Although it would be tempting to provide an exhaustive list, this is not possible, as new sets are being released every day. So we decided to focus on the most popular sets, with one additional condition. This is the availability of the set to be downloaded and the possibility to use it for research.

**Figure 3:**
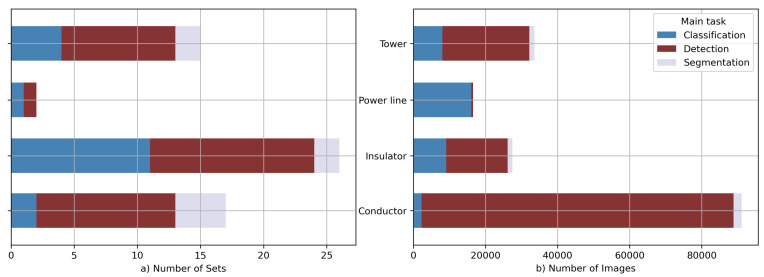
The summary statistics for the reviewed data collections. The objects of the specific classes have been aggregated into more general categories.

Table 1 provides a detailed list of the selected datasets and their references. We have indicated the main purpose of each dataset and attributed all the classes that are contained. For most of the datasets, we have also given the most important information, which should help to create some interest. A summary view of the reviewed datasets is depicted in Figure 3.

A number of the listed image batches could be used for classification problems [13,15,17,19,20,21,23,24,28,29,30,32,33,34,35]. There are also sets that provide ground truth labels for detection algorithms [12,14,15,16,17,18,19,20,21,22,23,25,26,27,28,29,30,31,32,33,34,35], but as can be easily noticed, some of the sets fit into both categories. There are also several that support segmentation tasks as well [22,26,31,33].

Some of the reviewed collections have been in the public domain for several years. This allows us to examine their impact on the field and verify that they really aim to provide working algorithms. We have listed the examples of such proofs of concept and published results in Table 2. These are investigated in the context of power line inspection, helping for instance in:

- robust defect analysis for power line equipment using convolutional neural networks (CNN), achieving, with this large evaluation dataset, up to 98% accuracy [36];
- preventing cascading failures of the grid [37];
- detecting power lines themselves using CNNs [26,38,39], or method based on epipolar constraints (PLAMEC) [40];
- transmission tower construction inspection (with detection of AP 89.9% [41]; or mAP of 94% using the ResNet50 framework [42]);
- for power line corridor monitoring with a mean average precision (mAP) of 72.45% from satellite imagery [27], or separation for vegetation management around power lines using time series analysis [43], or single images only [44];
- for sub-element diagnostics, such as conductor detection purposes (with AP of 0.729 [12]) or insulator detection [45].

**Table 2:** Summary of the main research achievements that were aided using the described sets.

Research Name	Research Results
Power Infrastructure Monitoring and Damage Detection Using Drone-Captured Images [46]	F-score of 75% for multi-class classification and 88% for pylon identification.
Detection and Monitoring of Power Line Corridor From Satellite Imagery Using RetinaNet and K-Mean Clustering [27]	mAP of 72.45% for an IoU threshold of 0.5 and 85.21% for IoU threshold of 0.3; discrimination of high- and low-density vegetation regions within the power line corridor area.
A Monocular Vision-Based Perception Approach for Unmanned Aerial Vehicle Close Proximity Transmission Tower Inspection [41]	Reported results: SSD300 network has the fastest runtime with 6 FPS, and YOLOv2 has a speed of 5.6 FPS, but their APs were relatively low—87.5 for SSD300 and 86.8 for YOLOv2. The highest AP (89.8) was obtained by Faster R-CNN (VGG16) and Tower R-CNN with 0.8 and 5 FPS, respectively.
Wire Detection using Synthetic Data and Dilated Convolutional Networks for Unmanned Aerial Vehicles [12]	Evaluation of a few deep neural networks (FCNs, SegNet, and E-Net) on a publicly available USF dataset. Obtained results: AP = 0.729, F-Score = 0.688 for wire detection using dilated convnets to facilitate autonomous UAVs.
Time Series Analysis of Separation for Vegetation Management Around Power Lines Using UAV Photogrammetry [43]	Separation distance between the power line and the vegetation at any position.
Detecting Power Lines in UAV Images with Convolutional Features and Structured Constraints [26]	Evaluation using two datasets (PLDU and PLDM) for power line detection using UAVs. Obtained F1-scores: ODS (optimal dataset scale threshold) = 0.914 (PLDU dataset) and 0.888 (PLDM dataset).
TTPLA: An Aerial-Image Dataset for Detection and Segmentation of Transmission Towers and Power Lines [22]	The best average scores for the bounding box and mask are 22.96% and 15.72%, respectively.
Insulator Visual Non-Conformity Detection in Overhead Power Distribution Lines Using Deep Learning [16]	Accuracy of 92% for material classification, and 85% for defect detection; F1-score of 0.75.
Power Distribution Insulators Classification Using Image Hybrid Deep Learning [47]	Overall accuracy of 95% for the identification of non-conforming component classes.
Real-time Power Line Detection Network Using Visible Light and Infrared Images [38]	mIoU of 37.68% with processing speed 24 fps on UAV.
Detection of Power Line Insulators on Digital Images with the use of Laser Spots [45]	The method enables positive classification of insulators based on profile fragments. It reports the prediction quality measures for insulator detection accuracy of 0.961 and precision of 0.989.
Autonomous Aerial Delivery Vehicles, a Survey of Techniques on how Aerial Package Delivery is Achieved [48]	A comprehensive review of various power line domain applications.
Object Detection-Based Inspection of Power Line Insulators: Incipient Fault Detection in the Low Data-Regime [49]	The best result was obtained for insulator detection (mAP up to 0.90), while the mAP was relatively lower for other objects (disks, dumpers, or nests).
Automatic Power Line Inspection Using UAV Images [40]	RMSE for the point cloud of 0.233 m, and RMSE for the power line of 0.205 m.
Deep Learning-Based Framework for Vegetation Hazard Monitoring Near Powerlines [44]	The performance of powerline detection using aerial images, with precision and recall values of 0.821 and 0.762 (both for mAP@0.5), and 0.798 and 0.563 (mAP@0.5:0.95).
Deep Learning-Based Detection for Transmission Towers Using UAV Images [42]	mAP for transmission tower detection achieved using the Faster R-CNN model reached 90% using Inception V2 and 94% using ResNet50; and for the SSD model reached 84% and 92% with Inception V2 and ResNet50, respectively.
High-Accuracy Insulator Defect Detection for Overhead Transmission Lines Based on Improved YOLOv5 [50]	Average accuracy of the algorithm of 97.4% for insulator detection.
Robust Powerline Equipment Inspection System Based on a Convolutional Neural Network [36]	Real-time equipment detection with 93% recall and 92% precision, and defect analysis with up to 98% accuracy.
ICARUS: Automatic Autonomous Power Infrastructure Inspection with UAVs [51]	Multiple sensors integration; autonomous detection, tracking, and identification of infrastructure components automation.
The Implementation of a Convolutional Neural Network for the Detection of the Transmission Towers Using Satellite Imagery [39]	The final dataset consists of 4944 labeled satellite images; the highest performance obtained using the test set was an accuracy of 0.9676, a precision of 0.9522, and a recall of 0.9361.
Evaluation of Power Insulator Detection Efficiency with the Use of Limited Training Dataset [2]	AP at the level of 0.8 for 60 training frames. The main contribution of the work is the evidence that a limited training set, in this case just 60 training frames, could be used for object detection, assuming an outdoor scenario with well-defined conditions.

Applications that use images collected by UAVs are also popular because they allow the development of a well-performing algorithm. Another example is the scale enhancement pyramid network, which achieved an AP score of 81.5% for small object detection [52]. It is also due to the fact that one of the most practical and certainly popular ways of image acquisition is the use of unmanned aerial vehicles (UAVs). Such devices have been used for the preparation of datasets, e.g., [26,40,42,43,44,46,48,51,52]. Among other things, they helped to provide a power infrastructure classification model using deep neural networks that achieved an overall F-score of 75% for multi-class classification scenarios and 88% for pylon identification [46].

Another large group of reported applications deals with the detection of power line insulators, e.g., from aerial images (with an mAP of 0.90 [49]), indoor augmented photographs (accuracy of 92% for classification [16]), using attribute vector acquisition and design of hybrid classifier architectures (obtaining 95% overall accuracy [47]), or even insulator defect detection, with a score of 97.4% achieved on Improved YOLOv5 [50].

The essence of this study is detailed in Table 1, which describes the available data collections examined for this paper, and Table 2, which presents selected studies benefiting from these data. It should be noted that the primary focus of the collections is on the main elements of overhead power lines, such as conductors, towers, and insulators, since they are also the most frequently required interventions. From the point of view of maintenance management and the need for preventive measures to avoid failures in the transmission network, it is essential to develop appropriate datasets to support further research and development of algorithms, i.e., for detection or classification of:

- auxiliary equipment of overhead power lines:
  – clamps for attaching wires and auxiliary lines;
  – accessories intended for connecting clamps with insulators;
  – accessories for suspending insulator chains on poles and connecting multi-row chains;
  – protective equipment fixed at both ends of the insulator chains, designed to spread the electric field strength;
  – power line spacers used to fix bundle wires and ensure a constant distance between them;
  – tuned mass dampers (Stockbridge dampers);
  – protective marking (aviation obstruction signs, bird flight diverters);
  – de-icing and anti-icing equipment;
- detection of objects colliding with high-voltage lines:
  – branches and trees (problem of transmission corridors);
  – stork nests on poles.

Developing a domain data collection, which was the main goal for this review, is rather difficult, especially when it should target power industry equipment. Such a collection should allow one to deal with a narrow spectrum of problems, aid dedicated algorithm and application development, and encapsulate a narrow field of knowledge. Since the most interesting elements of the power line infrastructure are objects located in its vicinity, it often requires the data acquisition process to be realized on an upper section of the power poles. Training data recorded from the ground would be very different from that recorded during diagnostic flights. This creates an additional difficulty in the process of collecting good quality samples, as it usually necessitates flying a helicopter in the vicinity of the power line. Such an approach significantly increases the cost of dataset development and often excludes smaller organizations and individuals from the market. Another barrier to the creation of such domain datasets is the need for the expertise required to classify and assess damage to elements of towers or other power line equipment after data collection. As part of the review, the current state of available collections, their quality and usefulness from the perspective of power line diagnostics automation has been presented. In our opinion, the most valuable among the presented collections is the set from [21], although it is not characterized by the highest number of training samples, it has all the elements that affect the quality and usefulness of this type of set:

- Size: the collection should provide enough data to allow accurate power line diagnostics. The more data, the better the chance of detecting damage. However, for the mentioned set ([21]), its size is not at the top of the list, suggesting that following the size criteria alone could be ambiguous. Its size is relatively small compared to others, but it is currently the most numerous in terms of found and labeled damage.
- Diversity: The collection should include a variety of data, different power line conditions, and address realistic scenes. In this way, it should be possible to identify problems in a variety of conditions, which is crucial for effective power line monitoring. In the mentioned set we have emphasized different groups of insulators and their damage have been collected (including two classes of damage: broken insulator shell, and flashover damaged insulator shell).
- Data quality: The image material in the selected set should provide the highest possible resolution in its recording, which later will help the precise marking process. This is one of the most important aspects of a well-prepared collection, while incorrect or inaccurate annotations can be misleading.
- Generic nature: The method of registration partially disqualifies the usefulness of some sets for applications in diagnostic solutions based on air flights. In the promoted set, the acquisition was performed from the air, which refers to similar recordings during a scheduled diagnostic flight.

### 2.2. The Collection of Images of an Insulator Taken Outdoors in Varying Lighting Conditions with Additional Laser Spots

**Figure 4:**
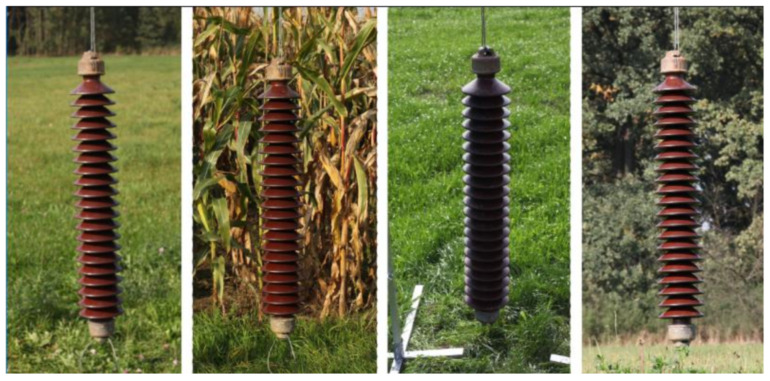
Collection of images of an insulator taken outdoors in varying lighting conditions with additional laser spots—research scenes.

There is one more special set that we would like to draw your attention to: the collection of images of an insulator taken outdoors in varying lighting conditions with additional laser spots [18]. The full information about its attributes is listed in Table 3. The developed dataset contains images of a long rod ceramic insulator made on several types of composite backgrounds. The backgrounds consist of scenes with a significant predominance of different shades of green, such as forest, farmland, and grass (Figure 4). The insulator was placed on a special construction that allowed images to be obtained in conditions close to reality.

**Figure 5:**
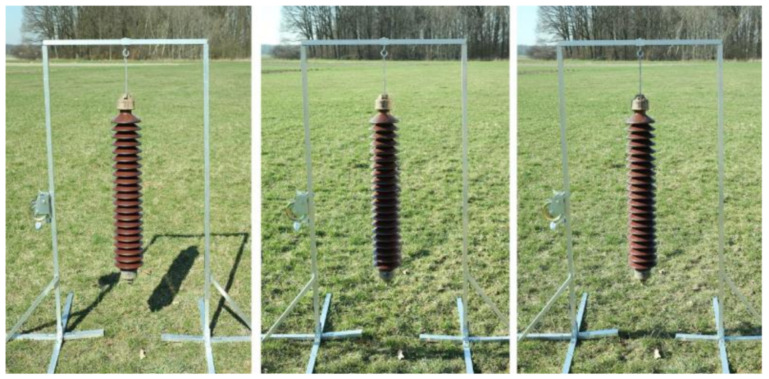
The setup that was used to develop the commented dataset, which was aimed at the acquisition of high-resolution images in varying lighting conditions with additional laser spots—samples with illuminance variation on the insulator surface are presented in this figure.

To evaluate the effect of lighting on the efficiency of the computational methods, the images were taken at different stages, at different times of day, and in different positions (horizontal and vertical). The position of the camera and the insulator were not changed during image acquisition. Examples of images taken under different lighting conditions are shown in Figure 5.

In order to obtain information about the variability in illumination on the surface of the insulator, the research had to be carried out outside the laboratory under natural lighting. The images of the insulators installed on the power lines are characterized by a great depth of image, caused by a considerable distance from the background elements. In order to achieve a similar effect, an aluminum structure has been constructed that allows the installation of various types of insulators with the possibility of smooth angle adjustment from 0 to 90°. The images were taken from a distance of 5 m using a Canon EOS 5D Mark II camera with Canon 50 mm f/1.8 and Tamron AF 28-300 mm lenses. During the registration, the light intensity was also recorded using the measuring instrument Testo 435-4 and the Testo 0635 0545 measuring instrument (lux probe for illumination level measurement). The change in intensity during the measurement was in the range of 632 to 45,479 lux. A dataset was prepared to verify the author’s method of detecting insulators using a high-power laser that generated a grid of points. In total, the collection contains 2630 images, with and without visible laser spots.

**Figure 6:**
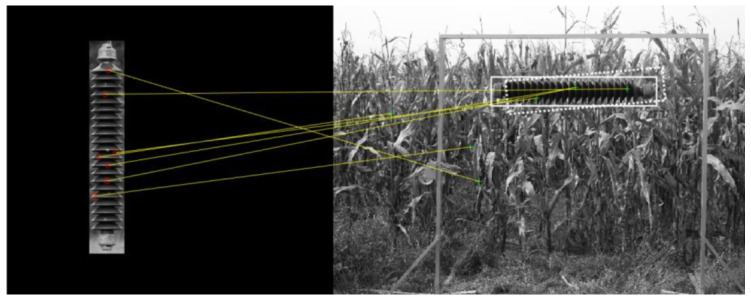
Example of the power line insulator detection attempt that was based on the analysis of local features.

The first application of the presented image collection was the development, testing, and evaluation of methods for detecting power line insulators on digital images. In order to accelerate the work on the algorithm, the process was supported by laser light, which allowed the area of the object search to be significantly reduced and the detection to be significantly speeded up. At the same time, other research aimed to develop new methods for detecting power infrastructure objects on high-resolution images in scenes with varying backgrounds. Figure 6 shows an example of using a local feature detector on a selected image from the dataset. Ref. [53] commented on the development of a dictionary of local features that would allow the detection and subsequent localization of damage in various types of power network elements on digital images taken during inspections carried out by flying devices (airplanes, helicopters, UAVs) or brigades and service vehicles on the ground. Another study showed the evaluation of profiles representing an insulator, characterized by regular patterns [45]. Using this dataset, it was verified that profiles extracted from these images could be accurately classified as containing or not containing an insulator. The achieved promising performance for the final insulator detection effectiveness was 0.989, while maintaining a precision of 0.979.

In addition, the presented dataset can be used to develop, verify, and compare:

- methods of detection and location of insulators in images;
- preparation of classifiers of technical facilities;
- methods of laser spot detection on digital images made outside in variable lighting conditions;
- methods of assessing the condition of the insulator’s surface, or other deep learning algorithms, in particular, convolutional neural networks, generative adversarial networks, or deep belief networks.

## 3. Conclusions

The presented review should be considered a source of such image sets as the ones reviewed, which could be used for the development of automated solutions for the power line industry, or as a blueprint for the process of preparing one’s own dataset.

A good example of such a collection is one of the most extensive of the presented datasets [21], which, although it is dedicated only to overhead line insulators, could be considered as a domain benchmark. Its main advantage and great value lies in the fact that it contains not only the position of a specific insulator, but also the information about its condition (broken/unbroken) for a variety of different types. Due to the rather rare occurrence of defects, such information is difficult to obtain, but it is also a highly sought-after feature.

With the rapidly growing number of unmanned devices equipped with high-resolution cameras, we are also seeing a significant trend in the number of datasets built from such imagery (described in Section 2.1). While UAVs are widely used to inspect overhead high-voltage lines and collect video, they are usually also the starting point for the creation of datasets. The latter use of them has also been developed. As shown in [26,38,40,42,43,46,51,52], this type of imagery can be used for many tasks that support the proper operation of the network infrastructure, which usually operates over a large area.

Most of the collections presented here are based on single modality data, in most cases an image from a camera or points from LiDAR sensors, which may not be sufficient for industrial solutions. Increasingly, however, the results of correct object detection and classification are based on data fusion from a group of various sensor modalities, such as VIS, IR, NIR, UV, and point clouds. Such datasets [54,55] are currently mainly used in the context of autonomous vehicle perception, but they show a certain trend in the use of multimodal data in conditions with high environmental variability. The proposed compilation of datasets can be used as part of the preparation of this type of multimodal collection as a preliminary classifier in the sample annotation process.

In the long term, the economic implications of the study conducted will allow the development of tools to optimize the planning and maintenance of critical infrastructure through the early identification of areas of increased degradation, which will directly lead to an increase in the life of the infrastructure. Undoubtedly, in the short term, the implementation of advanced diagnostic technologies may require significant investment, which is a challenge for operators and energy companies.

## Figures and Tables

**Table 1:** Overview of the main image datasets for power line elements. MT stands for the main task of the dataset, where C is for classification, D is for detection, S is for segmentation, and NoI reflects the number of images in the collection.

Name and Reference	Classes	MT	NoI	Details
Wire detection dataset [12]	Conductor	D	67,000	The set contains synthetic images. Top models were run at more than 3 Hz on the NVIDIA Jetson TX2 with an input resolution of 480 × 640, with an average precision score of 0.73.
Power Lines Detection (Recognizance-2) [13]	Power line	C	16,078	Contains images of power lines from a set of given visible and infrared images.
Aerial Power Infrastructure Detection Dataset [14]	Tower	D	12,943	The dataset consists of top-view images of MV poles from various locations across Cyprus. Images were captured across different seasons to account for a variety of background conditions, such as grass or ground, as well as at different heights to account for variations in the UAV’s height during the inspection. Additionally, all annotations were converted into VOC and COCO formats for training in numerous frameworks.
Powerline Image Dataset (Infrared-IR and Visible Light-VL) [15]	Conductor, No conductor	D	8000	The set contains IR and VL images that were acquired and scaled to a size of 128 × 128. Conductors and towers are present in images.
Overhead Power Distribution Lines Insulators dataset (OPDL Dataset) [16]	Insulator, Conductor	D	4960	In this database, 4 types of distribution insulators were selected that operate at a voltage of 15 kV, namely: ceramic pin insulator (CPI), ceramic bicolor insulator (CBI), polymeric grey insulator (PGI), and glass green insulator (GGI), taken inside and outside the studio.
Combine Pole Computer Vision Project [17]	Tower	C, D	3761	The dataset shows the upper element of the power pole in most cases, together with the insulators. Images were taken in different lighting conditions. There are also samples recorded at night.
Electrical insulators dataset [18]	Insulators	D	2630	Images of a long rod electrical insulator under varying lighting conditions and against different backgrounds: crops, forest, and grass. Images contain additional laser spots that could be exploited to boost object detection. Longer description in Section 2.2.
Power Towers Computer Vision Project [19]	Tower, Insulator, Conductor	C, D	2121	The set contains photos from many different sources. The labels describe mainly the supporting structures of high-voltage lines.
Electrical Substation Computer Vision Project [20]	Tower, Insulator	C, D	1991	A large part of the photo collection shows the power infrastructure in winter scenery, some with elements of overhead lines covered with ice or snow. The labels describe columns and supporting structures.
The Insulator Defect Image Dataset (IDID) [21]	Insulator, Flashover damage insulator shell, Brokeninsulatorshell, Unbrokeninsulator shell	C, D	1596	The images present the insulator as the primary subject and a parent class; each image is assigned to one of 3 sub-classes: flashover damage insulator shell, broken insulator shell, or good insulator shell. In addition, a text document is included with an overview of the dataset characteristics, file structure, and labeling format.
Transmission Tower Dataset in VOC format [22]	Tower, Conductor	D	1300	Collected from internet and inspection videos. Various types of towers and backgrounds.
TTPLA: An Aerial-Image Dataset for Detection and Segmentation of Transmission Towers and Power Lines [22]	Insulator, Conductor, Tower	S, D	1234	Images with a resolution of 3840 × 2160 pixels and manually labeled; a total of 8987 instances of transmission towers and power lines.
MNV0L Dataset [23]	Insulator, Broken insulator	C, D	931	Different types of insulators and their damage are included. There are no labels attached to this collection.
Insulator Dataset—Chinese Power Line Insulator Dataset (CPLID) [24]	Insulator, Broken insulator	C	848	The number of non-broken insulator images is 600. The number of defective insulator images is 248. Real-world images with labeled insulators are supplemented with synthetic images with defects labeled (i.e., missing cap).
Ground Truth of Powerline Dataset (Infrared-IR and Visible Light-VL) [25]	Conductor, Noconductor	D	800	The set contains 400 IR and 400 VL images that are acquired and scaled to a size of 512 × 512. The IR folder contains IR images with power lines, ground truths, and overlay images of these images. The VL folder contains VL images with power lines, ground truths, and overlay images of these images. Conductors and towers present on images.
Power line dataset of urban scene (PLDU) [26]	Conductor	S, D	573	The images in this dataset were captured with UAVs hovering above the power lines within ten meters. Images have pixel-wise annotations
Electric transmission and distribution infrastructure imagery dataset [27]	Power line, Tower	D	511	Dataset built of annotated electric transmission and distribution infrastructure for approximately 321 km^2^ of high-resolution satellite and aerial imagery, spanning 14 cities and 6 countries across 5 continents. This dataset was designed for training machine learning algorithms to identify electricity infrastructure in satellite imagery automatically; for those working on identifying the best pathways to electrification in low- and middle-income countries, and for researchers investigating domain adaptation for computer vision.
Insulator Final Computer Vision Project [28]	Insulator	C, D	498	The collection contains images of pin insulators from various manufacturers. The images show the insulators on very different scales (resolutions).
SEAI-C4 Computer Vision Project [29]	Insulator, Broken insulator	C, D	469	Dataset with various types of insulators, mostly made of glass and ceramic. The photos have noise added at the post-processing stage.
Power line dataset of a mountain scene (PLDM) [26]	Conductor	S, D	287	Dataset elements were captured at a distance of more than thirty meters of power lines in mountain scenery.
CEPS Computer Vision Project [30]	Insulator	C, D	257	Images of different types of insulators are depicted on very diverse backgrounds.
Power line vegetation management using UAV images [31]	Conductor, Tower	S, D	187	An example dataset containing UAV images for power line vegetation encroachment detection. The images contain GPS positions in their EXIF metadata.
PLAD: A Dataset for Multi-Size Power Line Assets Detection in High-Resolution UAV Images [32]	Transmission tower, Insulator, Spacer, Tower plate, Stockbridge damper	C, D	133	Image size: 5472 × 3078 or 5472 × 3648. It has 2409 annotated objects divided into five classes, which vary in size (resolution), orientation, illumination, angulation, and background.
Broken glass insulator Computer Vision Project [33]	Insulator, Broken insulator	C, D, S	125	Images depicting glass insulators. Most of the labels are rectangular in shape, and part of them indicate the exact outline of the insulator and are suitable for segmentation.
Electrical Line Computer Vision Project [34]	Conductor	C, D	120	Insulated and non-insulated wires are labeled separately, although the markings are not very precise.
Dataset Insulators Neering Computer Vision Project [35]	Insulator, Broken insulator	C, D	104	The dataset contains labels of faults located on the surface of insulators.

**Table 3:** Detailed list of presented power line insulators dataset components.

Element	Data Format	Description
Digital images of power line insulators	JPG	Images of the insulator on a different background.
Digital images of power line insulators with laser points	JPG	Images of insulator on a different background along with visible points of a green laser generated by a device emitting light with a wavelength of 532 nm.
ROI	CSV	Information on the position of the insulator in the ROI picture (x, y, width, height).
Illuminance	CSV	Information on the lighting level in lux (lx) at the moment of taking a given image.
Position of laser points on images	CSV	For images on which laser spots are located, information about their number and the coordinates of the spots in the image.

## Data Availability

The investigated dataset of power line insulators images can be accessed here [18]. Links to the other described datasets are provided in the references section.

## Abbreviations

The following abbreviations are used in this manuscript:

AP	Average precision
COCO	Common Objects in Context, a dataset for object detection
CPLID	Chinese Power Line Insulator Dataset
CNN	Convolutional neural network
DNN	Deep neural network
EXIF	Exchangeable image file format; image metadata standard
fps	Frames per second; indicates frame rate
GPS	Global positioning system
IDID	Insulator Defect Image Dataset
IoU	Intersection over union metric
IR	Infrared

LiDAR	Light detection and ranging
mAP	Mean average precision
mIoU	Averaged IoU metric
MV	Medium voltage
NIR	Near infrared
nm	Nanometer; 1 nm = $10^{-9}$ m
OPDL	Overhead Power Distribution Lines Insulators dataset
PLAD	Power Line Assets Detection Dataset
PLDU	Power line dataset of urban scene
PLDM	Power line dataset of mountain scene
R-CNN	Region-based convolutional neural networks
ResNet50	50-layer residual convolutional neural network
RMSE	Root mean square error
TTPLA	Transmission Towers and Power Lines dataset
UAV	Unmanned aerial vehicle
UV	Ultraviolet
VOC	The PASCAL Visual Object Classes 2012 dataset
VL	Visible light
YOLOv5	5th version of you only look once object detection network
